# Improving fluorescence lifetime imaging microscopy phasor accuracy using convolutional neural networks

**DOI:** 10.3389/fbinf.2023.1335413

**Published:** 2023-12-22

**Authors:** Varun Mannam, Jacob P. Brandt, Cody J. Smith, Xiaotong Yuan, Scott Howard

**Affiliations:** ^1^ Department of Electrical Engineering, University of Notre Dame, Notre Dame, IN, United States; ^2^ Department of Biological Sciences, University of Notre Dame, Notre Dame, IN, United States

**Keywords:** phasor lifetime synthesis, fluorescence lifetime imaging microscopy (FLIM), lifetime image analysis, convolutional neural networks (CNNs), phasor clustering method, image segmentation, deep learning

## Abstract

**Introduction:** Although a powerful biological imaging technique, fluorescence lifetime imaging microscopy (FLIM) faces challenges such as a slow acquisition rate, a low signal-to-noise ratio (SNR), and high cost and complexity. To address the fundamental problem of low SNR in FLIM images, we demonstrate how to use pre-trained convolutional neural networks (CNNs) to reduce noise in FLIM measurements.

**Methods:** Our approach uses pre-learned models that have been previously validated on large datasets with different distributions than the training datasets, such as sample structures, noise distributions, and microscopy modalities in fluorescence microscopy, to eliminate the need to train a neural network from scratch or to acquire a large training dataset to denoise FLIM data. In addition, we are using the pre-trained networks in the inference stage, where the computation time is in milliseconds and accuracy is better than traditional denoising methods. To separate different fluorophores in lifetime images, the denoised images are then run through an unsupervised machine learning technique named “K-means clustering”.

**Results and Discussion:** The results of the experiments carried out on in vivo mouse kidney tissue, Bovine pulmonary artery endothelial (BPAE) fixed cells that have been fluorescently labeled, and mouse kidney fixed samples that have been fluorescently labeled show that our demonstrated method can effectively remove noise from FLIM images and improve segmentation accuracy. Additionally, the performance of our method on out-of-distribution highly scattering in vivo plant samples shows that it can also improve SNR in challenging imaging conditions. Our proposed method provides a fast and accurate way to segment fluorescence lifetime images captured using any FLIM system. It is especially effective for separating fluorophores in noisy FLIM images, which is common in in vivo imaging where averaging is not applicable. Our approach significantly improves the identification of vital biologically relevant structures in biomedical imaging applications.

## 1 Introduction

In addition to conventional fluorescence imaging, fluorescence lifetime imaging microscopy (FLIM) is a fundamental methodology in the biomedical imaging field that enhances the contrast of molecular structure. In light microscopy, FLIM is used to precisely measure the fluorescence decay lifetime of excited fluorophores (Chang et al., 2007; Mannam et al., 2020a; Datta et al., 2020). By doing this, FLIM provides a highly effective tool for researchers. This important metric indicates the average time the fluorophore remains in the excitation condition before returning to the ground condition. Remarkably, FLIM offers distinct perspectives on many biochemical factors, such as ion concentrations, concentrations of dissolved gases, refractive index, pH levels, and micro-environmental conditions within living samples, immune to sample excitation power, fluorophore concentration (either adhesive or auto-fluorescence), and photobleaching, all of which are often very difficult to control in the majority of the experiments (Datta et al., 2020). FLIM systems are broadly categorized into two types: time-domain FLIM (TD-FLIM) and frequency-domain FLIM (FD-FLIM). The TD-FLIM method involves the precise measurement of the time that elapses between the excitation of the sample by a pulsed laser and the photon arriving at the detector. In contrast, FD-FLIM exploits the modulation changes and the relative phase between the emitted fluorescence and the excitation using periodic modulated pulses for FLIM lifetime image generation.

**FIGURE 1:**
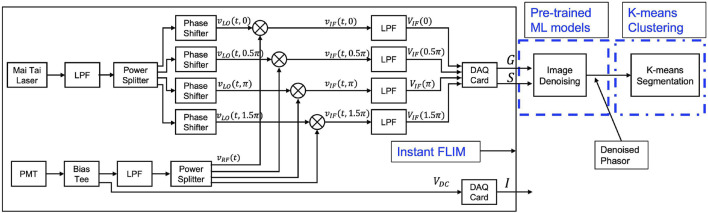
A schematic diagram of the Instant FLIM system (Zhang et al., 2021), which simultaneously extracts intensity and lifetime information from analog signals. *G* and *S* denote the real and imaginary components in the phasor space of the complex FLIM measurement. The *G* and *S* images are denoised utilizing our pre-trained image denoising model, which is marked as $\hat{G}$ and $\hat{S}$ . We obtain the denoised lifetime image by taking the ratio of $\frac{\hat{S}}{\hat{G}}$ (without considering the scaling factor). Pre-trained machine learning models, which were utilized for lifetime denoising and K-means segmentation algorithms to cluster fluorophores, are highlighted with a blue dotted box. Additional details about the experimental setup is explained in Section 2.1. PMT: photomultiplier tube, LPF: low-pass filter. Figure is derived from conference proceedings (Mannam et al., 2021).

Conventional FLIM systems (both TD-FLIM and FD-FLIM) are limited by slow processing speeds, a technical constraint; low signal-to-noise ratio (SNR), a fundamental constraint; and expensive and sophisticated hardware setup (Mannam et al., 2020a). We have developed a new Instant FLIM system (Zhang et al., 2021) that utilizes analog signal measurements for high-speed data collection, as illustrated in Figure 1, to overcome the technical limitations of the FLIM system. This approach eliminates bandwidth bottlenecks by incorporating pulse modulation with high-efficiency techniques and affordable deployment with readily available high-frequency devices such as mixers, low-pass filters, and phase shifters. Furthermore, the analog measurements of the down-converted frequency (intermediate frequency) signals used in our demonstrated frequency-domain Instant FLIM system enables the simultaneous measurement of fluorescence intensity, fluorescence lifetime, and frequency-domain phasors during 2-D, 3-D, or 4-D imaging for both live (*in vivo*), *in vitro* and fixed cell (*ex vivo*) applications.

Another fundamental problem is the low SNR, which remains to be addressed. Conventional image denoising techniques, such as averaging (mean filtering) within the same field-of-view (FOV) or median weight filtering, can enhance the SNR at the cost of image blurring and reduced frame rates with high computational time. Other image denoising method is block matching 3D filtering (BM3D) (Dabov et al., 2007) due to its ability to effectively remove noise from images while preserving image details. This method is well-suited if the images corrupted by the Gaussian noise. However, this BM3D image denoising method performance is low in the low-light condition typically for the *in vivo* imaging. Overall, each of these image denoising methods have trade-offs between denoising performance, computation speed, and applicability to a specific noise distribution. Offline image denoising methods can also be used to improve the SNR, but they require the data to be stored before and after denoising, which is not feasible for memory-intensive imaging modalities such as light-sheet microscopy (Mannam et al., 2020a). A potential solution to this problem is to use real-time machine learning models for lifetime image denoising. This approach has the potential to improve the low SNR problem without compromising instant FLIM’s real-time imaging capabilities.

Machine learning (ML) has become more popular in recent years for its ability to enhance image processing performance, particularly in the area of image denoising (Goodfellow et al., 2016; von Chamier et al., 2021; Weigert et al., 2018; Nehme et al., 2018; Mannam and Howard, 2023). Several machine learning (ML) methods have been successful at denoising images with Gaussian, Poisson, or mixed Poisson-Gaussian noise (Weigert et al., 2018; Mannam et al., 2022). However, most machine learning methods demand dedicated large training datasets for noise reduction in the images during the training stage. Additionally, pre-trained models are typically only effective for specific tasks, such as intensity image denoising. This paper presents the application of a pre-trained ML model (DnCNN Denoising) for phasor image denoising in FLIM measurements. The pre-trained DnCNN model was trained on a dataset comprising 12,000 images of fluorescence intensity. By utilizing the pre-trained machine learning models, researchers obviate the demand for the development of a new model exclusively for lifetime image denoising since the noise distribution of the complex FLIM measurement corresponds to the training dataset’s noise distribution called mixed Poisson-Gaussian (MPG) noise. Moreover, our DnCNN model achieves precise phasor denoising outcomes at a significantly faster processing speed than conventional lifetime denoising approaches, such as median filtering. The denoised phasor image is obtained from the denoised FLIM measurements, followed by segmenting the denoised phasor using an unbiased ML technique called K-means clustering. This segmentation separates each fluorophore accurately in the denoised phasor image compared to the noisy phasor image. Section 2 outlines both the pre-existing and our created pre-trained ML model techniques for lifetime image denoising. Section 3 reports the comprehensive results of our approach on various test samples, with both qualitative and quantitative metrics. Finally, Section 4 presents our conclusion.

## 2 Methods for fluorescence lifetime denoising

In this section, the traditional and machine learning methods used to perform lifetime image denoising are explored. The orthogonal axes of FLIM measurements (*G* and *S* measurements), as depicted in Figure 1, are utilized in our in-house developed instant FLIM system for the extraction of lifetime information through the difference between two complementary-phase mixers, presented in the subsequent equations.

$$\begin{array}{c} S\propto V_{\text{IF}}\left(0\right)-V_{\text{IF}}\left(\pi \right),G\propto V_{\text{IF}}\left(0.5\pi \right)-V_{\text{IF}}\left(1.5\pi \right) \end{array}$$ (1)

where, *V*_IF_(*ϕ*) indicates the intermediate frequency voltage at the given phase of *ϕ*.

Extracted lifetime is defined as, *τ*, as ratio of imaginary (*S*) to real (*G*) measurement at a given position using the equation *τ* = *S*/(*ω* ∗ *G*), ignoring the scaling factor. In the FD-FLIM system, the resulting lifetime is calculated using the following equation

$$\tau =\frac{1}{\omega }\frac{V_{IF}\left(0\right)-V_{IF}\left(\pi \right)}{V_{IF}\left(0.5\pi \right)-V_{IF}\left(1.5\pi \right)}$$ (2)

where *ω* = 2*πf*_mod_, and the laser pulse modulation frequency (*f*_mod_), is 80 MHz as implemented in our instant FLIM measurement setup. Phasor plots can be easier to interpret and visualize lifetime information in TD- and FD-FLIM measurements. This simplification is achieved by transforming the fluorescence lifetime value of each pixel into a point within a 2-D phasor plot. The *x*-coordinate is represented by the coordinate *g*, while the *y*-coordinate is represented by the coordinate *s*. Phasor coordinates (*g* and *s* pairs) are used to distinguish between different fluorophores and excited state reactions in a phasor plot. The phasors for the TD-FLIM time-correlated single photon count (TCSPC) system can be extracted using the transformations presented in the subsequent equations.

$$\begin{array}{c} g_{i}=\frac{\int_{0}^{\infty }I\left(t\right)\cos\left(wt\right)dt}{\int_{0}^{\infty }I\left(t\right)dt},s_{i}=\frac{\int_{0}^{\infty }I\left(t\right)\sin\left(wt\right)dt}{\int_{0}^{\infty }I\left(t\right)dt} \end{array}$$ (3)

where *I*(*t*) is the TCSPC fluorescence intensity information at the *i* − th pixel and time index of *t*. For the analysis of FD-FLIM system, the phasors are extracted through the calculation of the following equations.

$$\begin{array}{c} g_{i}=m_{i}\cos\left(\phi_{i}\right),s_{i}=m_{i}\sin\left(\phi_{i}\right) \end{array}$$ (4)

where *ϕ*_*i*_ and *m*_*i*_ represent the phase shift and modulation degree change of the emission in comparison with the high-frequency excitation, respectively, at a random *i* − th pixel. The resulting phasor plot enables the identification of groups of pixels with similar fluorescence decays, which facilitates image segmentation. Therefore, the clustering method aids in identifying fluorophores with similar lifetime decay values on the phasor plot belonging to a single cluster.

### 2.1 Traditional methods

**FIGURE 2:**
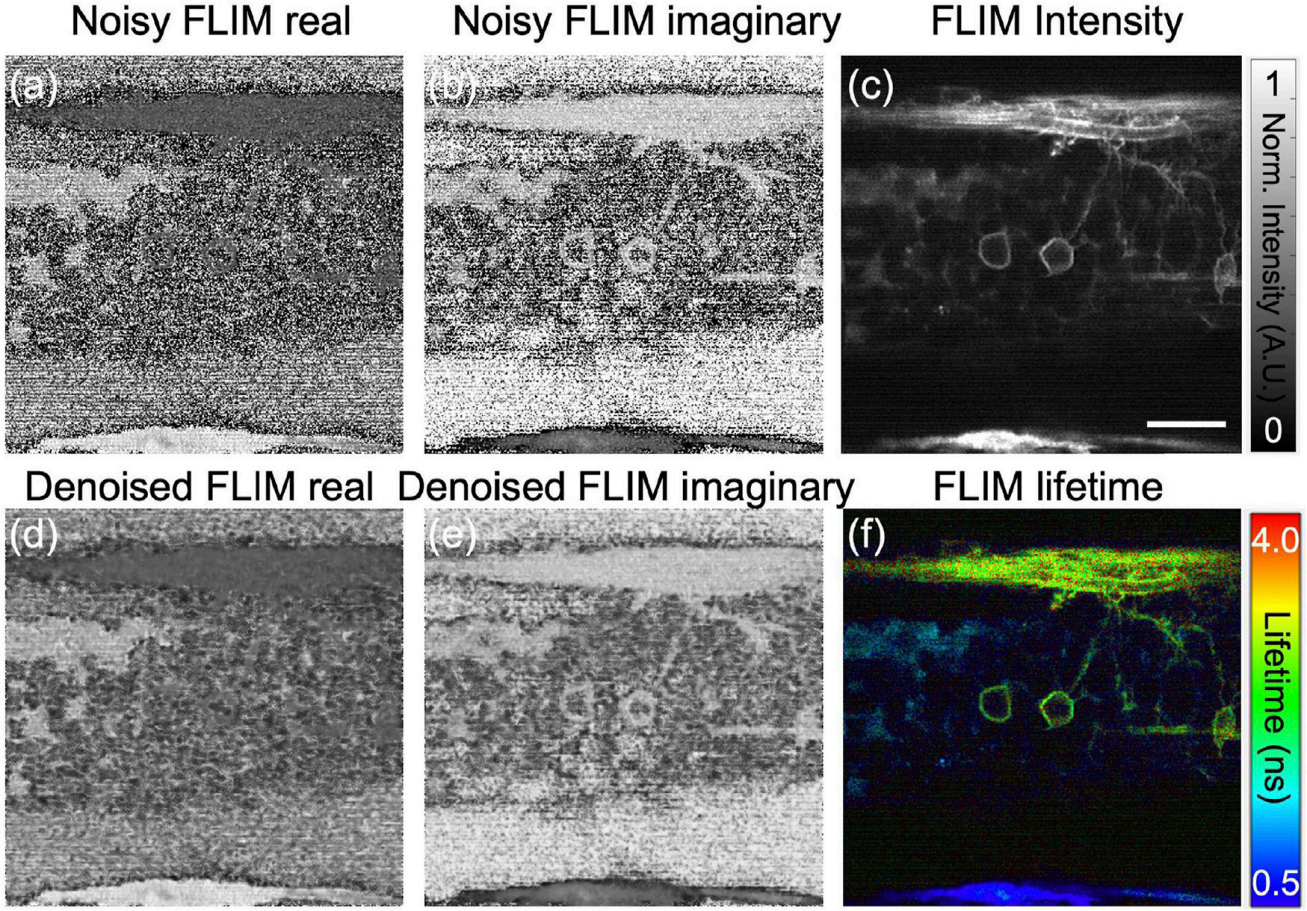
Using our custom-built instant FD-FLIM setup, the Noisy FLIM measurements were captured in an *in vivo* zebrafish embryo [*Tg(sox10:megfp)* at 2 days post-fertilization] in **(A)** and **(B)** for both *G* and *S*, while denoised images can be found in **(D,E)**, respectively. Fluorescence intensity **(C)** and composite lifetime **(F)** images are provided, respectively. The composite lifetime corresponds to the hue saturation value (HSV) representation of the intensity and lifetime images combined, where the pixels’ brightness and hue are utilized to map the fluorescence intensity, and lifetimes, respectively. Excitation wavelength of 800 nm with a sample power of 5.0 mW and pixel dwell-time of 12 *μ*s. To improve signal-to-noise (SNR), a 3D-volume of size 360 × 360 × 48, with each slice depth measuring 1 *μ*m across 48 slices and a pixel dwell time of 12 *μ*s, was averaged three times. FLIM Intensity image is normalized between 0 to 1 as shown in **(C)**. Scale bar: 20 *μ*m. Figure and data are derived from conference proceedings (Mannam et al., 2021).

Typically, FLIM measurements are significantly noisy for *in vivo* imaging at low excitation power, and hence the phasor plot, potentially representing inaccurate clustering of sample boundaries to identify underneath fluorophores. One approach for phasor noise reduction in complex FLIM phasor axes is through conventional filtering methods like mean and median filters. However, these filters must be iteratively applied more than once to effectively reduce the noise and achieve a high SNR. Median filtering preserves edges in denoised image relative to mean filtering (Digman et al., 2014), it requires multiple runs on the FLIM measurements (both orthogonal real and imaginary planes) to reduce the phasor noise. To illustrate this feature, we show the phasor of the 3-D volume of *in vivo* zebrafish embryo and perform the median filter a couple of times on the orthogonal real and imaginary axes of complex FLIM measurements. Figure 2A, B show the noisy FLIM measurements (both real:*G* and imaginary:*S* axes of complex FLIM measurements, respectively) of an *in vivo* zebrafish embryo captured with our in-house customer made instant FLIM system and the system data processing is depicted in Figure 1. Figures 2C, F show the fluorescence intensity and composite lifetime, respectively. The composite lifetime corresponds to HSV (hue-saturation-value) representation of the fluorescence intensity and fluorescence lifetime images combined, where the pixels’ brightness and hue are utilized to map the intensity, and lifetime values, respectively. To illustrate, we present a single plane from the 3D-volume stack, exhibiting neural cells fluorescence marked with an enhanced green fluorescent protein (EGFP) with lifetime ranging from approximately ≈1.5–2.5 ns. The low-lifetime region, approximately(≈0 ns–0.5 ns), signifies the presence of neural cells in the spinal cord. Figure 2.

**FIGURE 3:**
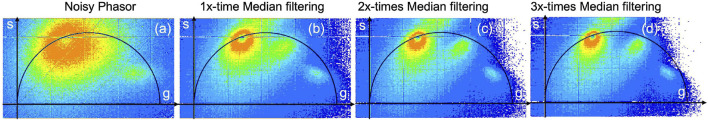
The raw phasor **(A)** and three iterations of the median filter [applied once **(B)**, twice **(C)**, and three times **(D)**] were applied to the complex FLIM measurements of *G* and *S* images of the *in vivo* zebrafish embryo *Tg (sox10: megfp)* at 2 days post-fertilization obtained using our custom-built instant FD-FLIM setup, respectively. In addition, for raw FLIM measurements refer to Figure 2, which includes both intensity and composite lifetime information. Figure and data are derived from conference proceedings (Mannam et al., 2021).

Phasor plots are obtained as a 2-D grid image in the *XY* plane, with real and imaginary FLIM measurement values projected onto the *x* − and *y* − axes, respectively. More details about phasor plots can be found in our lab’s previous review paper, Mannam et al. (2020a). Figure 3A illustrates the zebrafish 3-D volume noisy phasor. Subsequent to this, Figure 3B–D demonstrate median filter (Digman et al., 2014) applied once, twice, and three times to reduce the noise of the phasors on the complex FLIM phasor, respectively. The phasor plot shown in Figure 3A cannot reveal the lifetime distribution corresponding to the EGFP neural cells (≈1.5–2.5 ns) due to noisy measurements. Conversely, the application of a median filter to complex FLIM measurement enables identification of the neural cells in the median-filtered phasor plot. In addition, applying median filtering more than once (“twice” or “thrice”) to complex FLIM measurements results in neural cells with improved SNR in the phasor. However, applying the median filter four or more times has no additional advantage and lacks in slow computational time. Table 1 indicates the median filtering on complex FLIM measurement of 3-D volume zebrafish sample’s execution time. From Table 1, performing median filtering more often iteratively on the phasor extracted from raw FLIM measurements is significantly intensive in computation time and not effective for accurate identification of lifetime regimes and hence fluorophore boundaries. (Figure 3; Table 1).

**TABLE 1:** Computation time for median filtering on FLIM measurement data of real *G* and imaginary *S* components of entire 3D volume stack in MATLAB (MATLAB, 2019) including the average value and standard error for the measurement presented before and after the ± symbol, respectively. In this case, the 3D volume of sample data with size 360 × 360 with 48 images in the volume stack. In addition, for raw FLIM measurements refer to Figure 2, which includes both intensity and composite lifetime information.

	3-D volume name	Execution time average ± standard error (seconds)
Median filter	G_volume	0.276 ± 0.0027 (once)
		0.355 ± 0.0022 (twice)
		0.432 ± 0.0025 (three times)
	S_volume	0.281 ± 0.0026 (once)
		0.347 ± 0.0021 (twice)
		0.423 ± 0.0024 (three times)

### 2.2 Pre-trained ML models

In this section, we demonstrate a unique pretrained CNN model trained on a diverse set of fluorescence microscopy intensity images utilizing CNNs for instantaneous denoising of both the orthogonal dimensions of the complex phasor axes (*G*, and *S* images) through the use of the FIJI tool, an image processing software (Rueden et al., 2017). The computation time for the image denoising is approximately ≈80 ms (low computation time is important for real-time applications) using a single image (Mannam et al., 2022) of size 512 × 512 pixels. Convolutional neural networks (CNNs) are a popular type of machine learning model used for computer vision tasks that involve image data. They are designed to achieve high accuracy and efficiency by analyzing the image’s features at different levels of abstraction. In this part of the subsection, we provide details on the development of pre-trained models. A CNN typically consists of an encoder block followed by a decoder block (Mannam and Kazemi, 2020). The encoder module decreases the size of the noisy input image by identifying the significant features and rejecting the unwanted noise. The decoder module restores noiseless images from encoders to their original size. Once the CNN model training using a large fluorescence microscopy image dataset is completed, the trained CNN model performance is evaluated on a test dataset. The FMD dataset, referred to as the “training dataset,” Mannam et al. (2022), comprises 12,000 raw fluorescence intensity images captured via multi-modal microscopes such as widefield, two-photon, and confocal microscopes. Our previous paper (Mannam et al., 2020b; Mannam et al., 2022) provides further information concerning the FMD dataset’s collection process, pre-processing and post-processing steps, and the ML model training process.

In our research, we utilized two pre-trained machine learning models specifically designed for denoising fluorescence images (*G* and *S* of complex phasor components) in the presence of mixed Poisson-Gaussian (MPG) noise. The first model, known as the “DnCNN,” is a supervised deep convolutional neural network (CNN) constructed using the DnCNN architecture (Zhang et al., 2017). This model excels at accurately estimating noise residuals present in noisy fluorescence real and imaginary components. Subtracting the estimated residuals from noisy inputs, the DnCNN model architecture generates denoised images that exhibit superior performance. The “Noise2Noise model,” the second model discussed (Lehtinen et al., 2018), operates on a self-supervised basis. It uses two noisy images as both input and target data, which are extracted in the same field-of-view (FOV). This approach is particularly helpful when obtaining ground truth data is challenging or impossible, such as *in vivo* imaging. Unlike the DnCNN model, the Noise2Noise training uses the noisy image captured as the target image in the same FOV. Our prior research details the training and testing procedures for these models, as outlined in Mannam et al. (2022). Notably, our investigation shows the effectiveness of these pre-trained models on out-of-distribution samples obtained from various resources. These evaluations encompassed various dimensions, including fluorescence intensity images captured through the Widefield2SIM microscope, diverse sample and structure types, varying levels of noise, different microscope modalities like dark-field microscopy, and fluorescence images in three-dimensional volume stacks. Our pre-trained models have demonstrated superior denoising capabilities in comparison to traditional denoising methods and existing machine learning-based fluorescence microscopy denoising models, as consistently observed through our testing. The pre-trained CNNs used in the following sections are publicly available and can be accessed through GitHub folder^1^.

Table 2 shows summary of the samples collected to demonstrate our approach using different FLIM systems and different samples to generate fluorescence microscopy denoised images and denoised phasor images. To use pre-trained ML models for images from a different distribution than the training dataset, it is important to analyze the noise distribution of the input images (*G* and *S*). Figure 1 shows the extraction of the complex phasor orthogonal axes from the FLIM measurement using the difference between two complementary phases: *G* = *V*_IF_(0.5*π*) − *V*_IF_(1.5*π*) and *S* = *V*_IF_(0) − *V*_IF_(*π*). The FLIM measurement signal, *V*_IF_(*ϕ*) originates from a photomultiplier tube (PMT) and other analog device components including low-pass filters (LPFs), which results in a noise distribution that contains both Poisson and Gaussian noise, also known as mixed Poisson-Gaussian (MPG) noise. The real (*G*) and imaginary (*S*) images of the FLIM measurement follow a Skellam distribution, which is the difference between two Poisson distributions (contributors 2004; Griffin, 1992). For large signal values, the Skellam distribution approximates a Gaussian distribution (Wang et al., 2021) with mean as the difference between the two *V*_IF_ measurements and variance as the sum of the variance of each *V*_IF_ channel. In this paper, we use pre-trained image denoising CNN models trained on MPG noise (in the FMD dataset) to denoise both axes of a FLIM measurement. During inference, we input the noisy real-axes (as shown in the *G* image) and imaginary-axes (as shown in the *S* image) to the pre-trained ML models to obtain the denoised complex FLIM measurements as $\hat{G}$ and $\hat{S}$ , respectively. Table 2.

**TABLE 2:** List of the samples experimentally captured to demonstrated our denoising approach along with experimental conditions for the reproduction of the sample images. Please note that for all of these samples are imaged using two-photon FD-FLIM systems with the two-photon excitation wavelength of 800 nm.

Sl. No	Sample name	Imaging type	Fluorophores labeled	Sample power (mW)	FLIM setup
1	Zebrafish	*in vivo*	EGFP	5	Instant FLIM
2	Mouse Kidney	*in vivo*	Auto fluorescence	5	Commercial FLIM
3	BPAE cells	*ex vivo*	DAPI, Alexa Fluor 488 phalloidin, MitoTracker Red CMXRos	5	Instant FLIM
4	Mouse Kidney	*ex vivo*	DAPI, Alexa Fluor 488 wheat germ agglutinin, Alexa Fluor 568 phalloidin	3.3	Instant FLIM
5	Plant images	*in vivo*	Auto fluorescence	3 & 5	Instant FLIM

**FIGURE 4:**
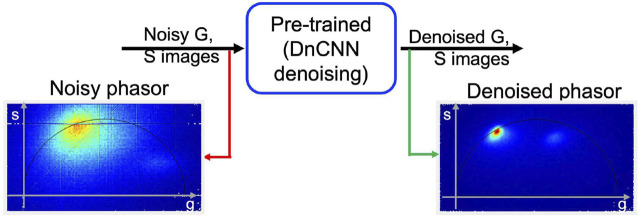
A block diagram illustrating the proposed methodology applied to the 3D volume stack depicted in Figure 2. The phasor representation before (shown in red arrow) and after (shown in green arrow) FLIM measurement denoising (noisy and denoised phasors) using our pre-trained DnCNN denoising model. Figure and data are derived from conference proceedings (Mannam et al., 2021).

Figure 4 depicts the proposed workflow for utilizing pre-trained ‘DnCNN’ machine learning models to denoise FLIM measurements and extract the phasor from the resultant denoised complex FLIM measurements. The method includes pre-processing and post-processing steps to restrict the range of orthogonal phasor axes to use the full-dynamic range of demonstrated pre-trained image denoising models and convert them back to their original scale during the inference process. Figures 2D, E display the denoised images of *G* and *S*, respectively, for one imaging plane in the 3D volume stack. Additionally, Figure 4 illustrates noisy phasors and denoised phasors through the use of our pre-trained DnCNN ML model. Figure 4.

To quantitatively assess the performance of lifetime image denoising methods, we employ the phasor representation of the fluorophores (Digman et al., 2014), the peak signal-to-noise ratio (PSNR) and the structural similarity index measure (SSIM) metrics (Gabriel, 2008; Sage, 2017). Phasor representation provides an accurate measurement of the fluorophore lifetime value and this measurement is independent of the excitation laser power, fluorophore concentration and only depends on the micro-environment. PSNR measures the mean squared error (MSE) between a denoised image and its corresponding clean reference image. The PSNR between a denoised image (*X*) and its corresponding clean (or averaged) image (*Y*) within the same field-of-view (FOV) is defined as: $\text{PSNR}\left(X,Y\right)=10\log(\frac{\text{max}{\left(Y\right)}^{2}}{\text{MSE}\left(X,Y\right)})$ , where $\text{MSE}\left(X,Y\right)=\frac{1}{N}\sum_{n=1}^{N}{\left(X_{n}-Y_{n}\right)}^{2}$ is the average mean-square error of *X* and *Y* with *N* pixels. SSIM evaluates the similarity between two images based on their luminance, contrast, and structural features. It produces a score between 0 and 1, where 1 indicates perfect similarity. Additionally, SSIM has been shown to correlate well with PSNR (Hore and Ziou, 2010). The PSNR and SSIM metrics are computed for the lifetime images after scaling them to 8-bit images using min-max normalization. The corresponding values are displayed in the scale bar of each fluorescence lifetime image.

While quantitative metrics like PSNR and SSIM cannot be calculated due to the lack of an averaged image, the phasor approach provides a qualitative assessment of the denoising effectiveness of pre-trained CNNs in *in vivo* zebrafish embryos FLIM measurements.

Finally, the segmentation in FLIM measurements that represents different fluorophores with accurate lifetime values (dividing into segments) is crucial for identifying fluorophores location of various lifetime values. The location of the fluorophores and their lifetime values are unknown in intravital imaging, where autofluorescence dominates. In a phasor plot, phasors with similar fluorescence decays cluster together, resulting in the need for accurate segmentation. Segmentation of the phasor occurs by selecting a cluster representing fluorophores with similar decay rates or lifetimes and assigning distinct colors to these clusters. The resulting images display the respective fluorophores using each color. To perform segmentation, typically a region marked with different colors is selected by the user in the phasor, and lifetime information is used to identify the fluorophore that belongs to this region. However, this method is prone to time consumption and unreliable outcomes, is contingent upon the user’s region selection, and cannot be reproduced. To prevent biased segmentation results, we suggest a fresh, impartial process for automatic phasor labeling, utilizing the unsupervised method named “K-means clustering” (Zhang et al., 2019). This algorithm finds consistent *K* centroids in a phasor plot and assigns each fluorophore to a cluster whose centroid is closest within a specific radius (MacQueen et al., 1967; Jain, 2010). K-means clustering automatically organizes the denoised phasor into precise clusters. The following section will discuss the results of the segmentation achieved through the utilization of this denoised phasor obtained using the pre-trained ML models.

## 3 Results and discussion

We applied pre-trained ML models to denoise FLIM data, resulting in clean phasors. These denoised phasors were further processed using K-means clustering across various test samples, as shown in the below sub-sections.

### 3.1 *In vivo* samples with phasor-segmentation

**FIGURE 5:**
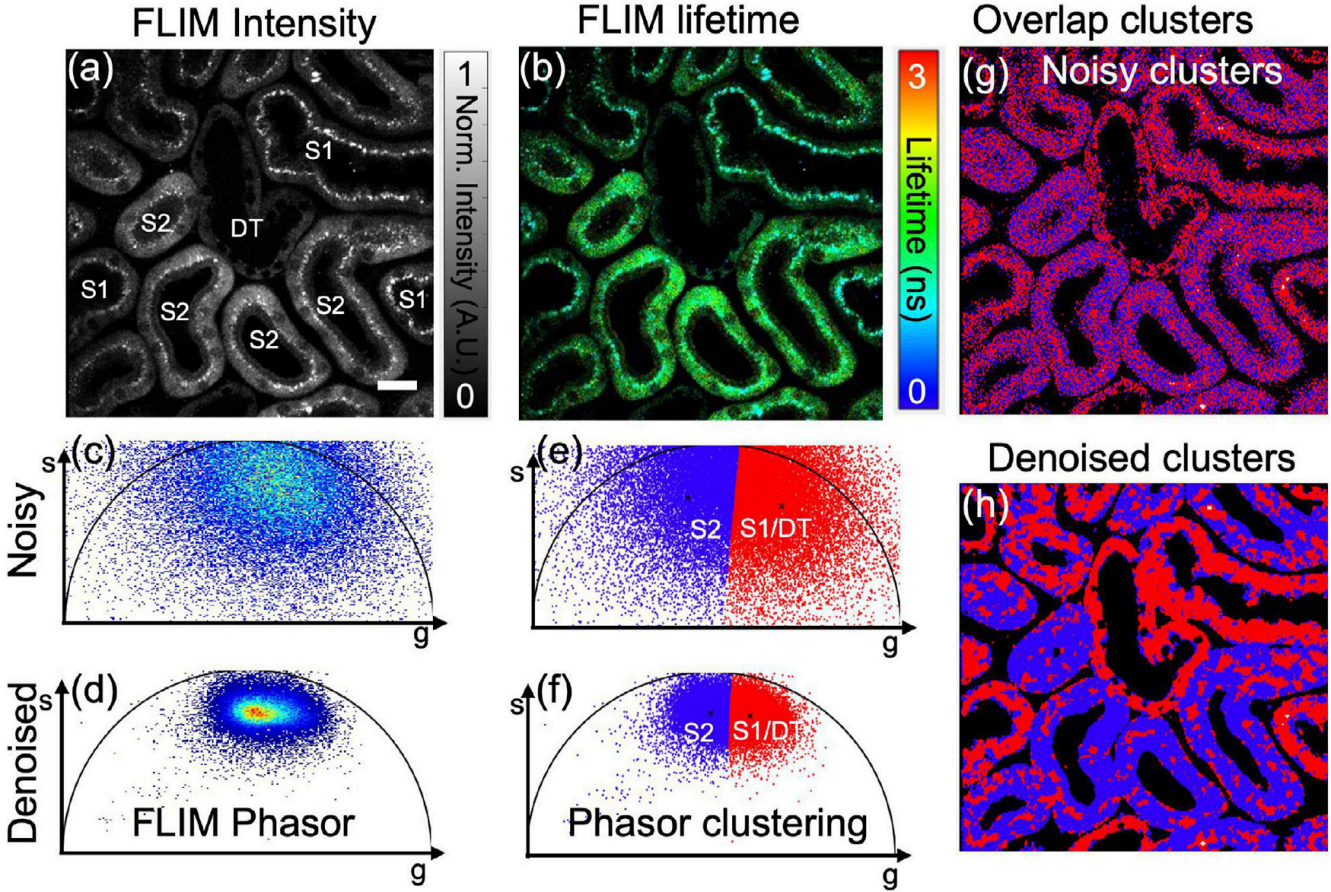
Application of K-means clustering segmentation on *in vivo* mouse kidney phasor data obtained through a commercial FD-FLIM system (Zhang et al., 2019). Panel **(A)** showcases a two-photon FLIM intensity image featuring microtubules indicating either *S*1/*DT* or *S*2, while panel **(B)** displays the corresponding fluorescence lifetime image. Phasor plots, denoted as **(C)** for noisy and **(D)** for denoised versions using our proposed method, are displayed. Additionally, K-means clustering on phasors is demonstrated in panels **(E,F)** before and after denoising, respectively. The microtubules *S*1/*DT* and *S*2 exhibit distinct lifetimes, represented by red and blue colors. The overlap of segment1 (*S*1/*DT*) and segment2 (*S*2) using noisy and denoised phasors is depicted in panels **(G,H)**, respectively. Excitation wavelength of 800 nm with a sample power of 5.0 mW and pixel dwell-time of 12 *μ*s. FLIM Intensity image is normalized between 0 to 1 as shown in **(A)**. Scale bar: 20 *μ*m. Figure and data are derived from conference proceedings (Mannam et al., 2021).

Figure 5 To illustrate our proposed pre-trained ML models for the FLIM lifetime image denoising, an *in vivo* mouse kidney sample is chosen and captured under a commercial FLIM setup. Figures 5A, B illustrate the fluorescence intensity and lifetime, respectively, for an *in vivo* mouse kidney (sample taken from The Jackson Laboratory, a male variant C57BL/6J mice of age at 8–10 weeks) acquired using a commercial FD-FLIM digital system (Zhang et al., 2019). False-color composite HSV images of fluorescence lifetime are shown in Figure 5B, where pixel brightness represents fluorescence intensity and hue represents fluorescence lifetime in the range of 0–3 ns. The fluorescence intensity image was labeled with the mouse proximal tubules (both upstream marked as *S*1 and downstream marked as *S*2) and distal tubules (*DT*), each having unique metabolic representations that are distinguishable through FLIM phasors. FLIM denoising is carried out on the complex FLIM phasor plane (both orthogonal real and imaginary axes) via our pre-trained ML models to extract denoised complex FLIM measurements represented as $\hat{G}$ and $\hat{S}$ , respectively. This process results in the extraction of an accurate phasor from denoised images. Figure 5C displays a single image of the phasor labeled for both upstream and downstream tubules obtained from the denoised phasor. Additionally, we observe that *S*1 upstream proximal tubules and *DT* distal tubules have similar lifetime distributions, although they differ morphologically, leading us to categorize them into only two clusters: *S*1/*DT* combined as one cluster and *S*2 as the other cluster. Figure 5E demonstrates an unsupervised cluster segmentation method named “K-means clustering” technique on a 2-D phasor plot. The K-means clustering findings reveal that color-coded red and blue color pixels in the noisy phasor correspond to *S*1/*DT* microtubules together as one cluster and *S*2 microtubules as another cluster, respectively. Notably, the proximal tubules of upstream tubules lying on the phasor’s right side (red color) have a shorter lifetime than the downstream tubules lying (blue color) on the phasor’s left side, which have a longer lifetime. However, the overlapping segmented clusters shown in Figure 5G make tubule identification challenging.

To resolve the problem of noisy FLIM measurements, we performed the pre-trained DnCNN model to denoise the FLIM measurements (complex phasor plane). This improves the phasor SNR, which is demonstrated in Figure 5D, exhibiting a high SNR compared to the noisy phasor as shown in Figure 5C. Once the denoised phasor is extracted, the K-means clustering method is used to achieve precise segments for the two proximal tubules (*S*1/*DT* and *S*2), as displayed in Figure 5F. After segmentation and denoising, the overlapped segmentation image was generated and can be found in Figure 5H. The red and blue tubules displayed in the image correspond to the *S*1/*DT* proximal tubules combined as one cluster and the *S*2 proximal tubules as another cluster, respectively. Hence, the demonstrated pre-trained DnCNN model provides rapid and precise automated segmented clusters of fluorescence lifetime images obtained through *in vivo* FLIM measurements. In cases where FLIM measurements exhibit noise, pre-trained CNN-based FLIM phasor denoising and segmentation using K-means clustering methods can prove beneficial. Clustering has been found to be an effective approach for improving biological structure detection in biomedical imaging research applications. An example where our method can be used to accurately identify microtubules of an *in vivo* mouse kidney sample is shown in Figure 5H. The absence of an averaged image hinders the calculation of quantitative metrics like PSNR and SSIM, mirroring the situation in zebrafish embryo FLIM measurements. Despite this limitation, the phasor approach offers a valuable qualitative evaluation of the denoising efficacy of pre-trained CNNs in the context of *in vivo* mouse kidney FLIM measurements.

### 3.2 Phasor denoising and clustering in fixed fluorescence samples

In addition to the *in vivo* mouse kidney samples, we show our demonstrated pre-trained ML model for FLIM denoising on a fixed BPAE sample. We acquired noisy BPAE sample images (Invitrogen prepared slide #1 F36924 by FluoCells) using our in-house customized two-photon frequency-domain fluorescence lifetime microscopy system, as described in Zhang et al. (2021). The samples featured three fluorphores: MitoTracker Red CMXRos labeled mitochondria (lifetime range from 1.5 ns to 2 ns), Alexa Fluor 488 phalloidin labeled F-actin (lifetime of values greater than 2.8 ns), and DAPI labeled nuclei (lifetime range from 2 ns to 2.8 ns). Instant FLIM setup includes a Nikon 40× magnification objective lens [water immersion, working distance (WD) of 3.5 mm, numerical aperture (NA) of 0.8], and emission wavelengths from 300 to 700 nm were filtered and collected collectively. The system also includes a photomultiplier tube (PMT) and a transconductance amplifier (TA) for the conversion of emitted photons to voltage. Further specifics can be found in Zhang et al. (2021). To capture the BPAE samples, the fixed cells are excited with a two-photon excitation wavelength of 800 nm (equivalent one-photon system of excitation wavelength of 400 nm) and with 5 mW sample power, 12 μs pixel dwell time, and 200 nm pixel width in the imaging plane. Emission spectra for the three fluorophores in fixed BPAE cells are provided in^2^, and the emission by the three fluorophores is collected together in our Instant FLIM system (Zhang et al., 2021).

**FIGURE 6:**
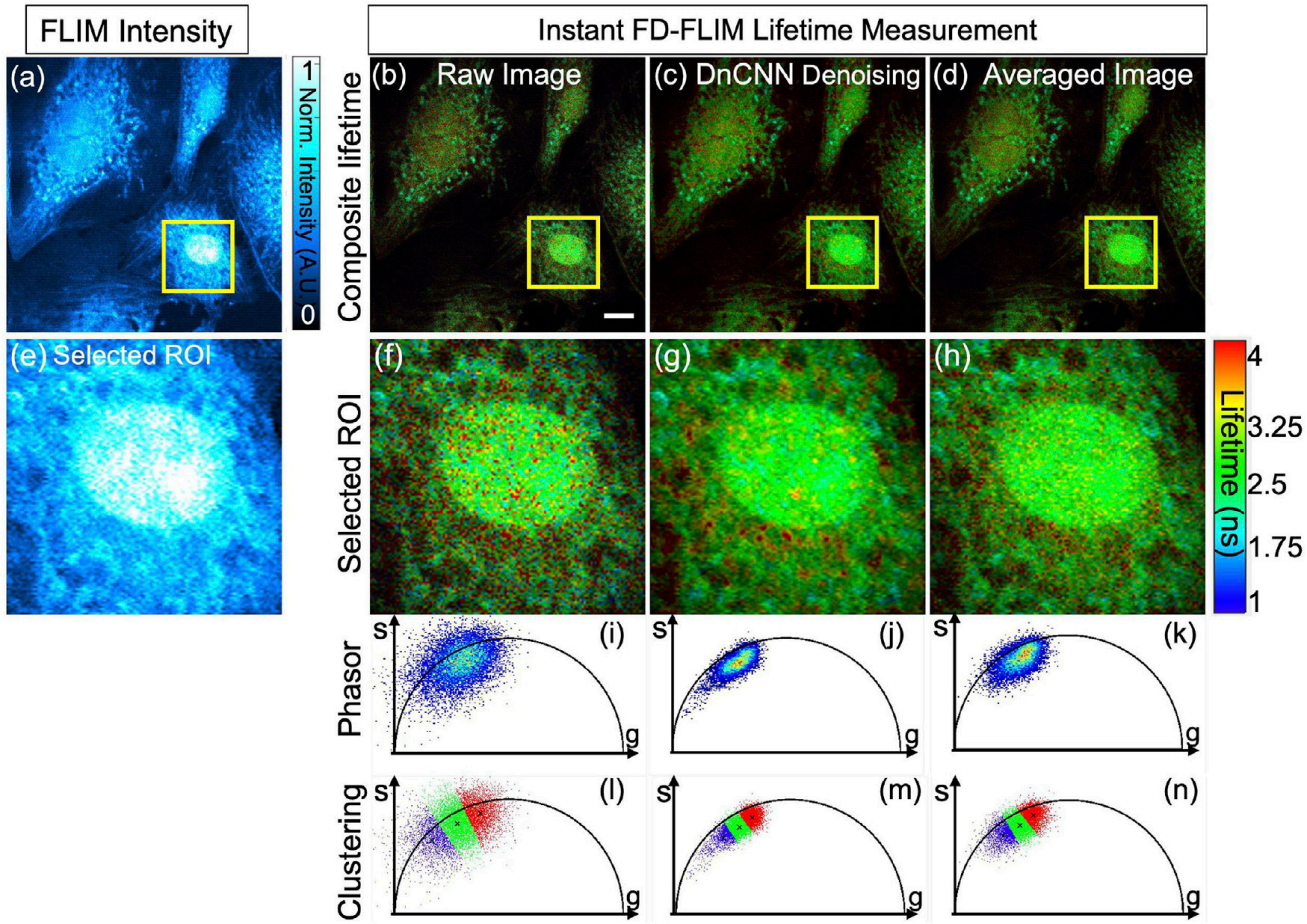
Phasor representation was obtained for a fixed BPAE sample using our instant FD-FLIM system, and pre-trained ML models were applied before and after. The FLIM data was acquired following (Zhang et al., 2021). The four images shown are **(A)** two-photon intensity, **(B)** noisy, **(C)** denoised using our proposed method, and **(D)** averaged fluorescence composite lifetime. Composite lifetime is represented in the form of hue saturation value (HSV) based on the combination of intensity and lifetime images, where the brightness of pixels is mapped to the intensity value while the fluorescence lifetimes are represented by the hue. The complete frame is of size 512 × 512 pixels while a small selected ROI is of 125 × 125 pixels. The intensity and composite lifetime of selected ROI for the noisy, denoised, and averaged images are illustrated in **(E–H)**, respectively. The phasors of the chosen ROI are displayed in **(I–K)** for the noisy, denoised, and averaged lifetime images, respectively. Red pixels indicate elevated lifetime values, which indicate noise in the combined lifetime images. K-means clustering (K = 3) for the noisy, denoised, and averaged lifetime images are displayed in **(L–N)**, respectively. The red cluster at **(L–N)** represents the nucleus of the BPAE cell in the selected ROI, where the fluorescence lifetime falls within the 2 ns**–**2.8 ns range. Conversely, the blue cluster indicates noise in the fluorescence lifetime image. Excitation wavelength is 800 nm, with the sample power at 5 mW and pixel dwell-time of 12 *μ*s. FLIM Intensity image is normalized between 0 to 1 as shown in **(A)**. The scale bar measures 20 *μ*m.

The qualitative outcomes obtained from the fixed BPAE sample are illustrated in Figure 6A, the noisy intensity of the fixed BPAE sample cell, acquired experimentally via the two-photon Instant FLIM system, is depicted (presented as a single-channel image using Cyan hot false color). Figures 6B–D display a composite lifetime image derived from the noisy FLIM data, denoised from the noisy FLIM BPAE data using a pre-trained DnCNN model, and averaged measurements (averaged lifetime over 5 acquisitions within the same FOV) representing high-SNR images for reference, respectively. Figures 6E–H show the selected ROIs for the intensity, composite lifetime images of noisy, DnCNN-denoised, and averaged lifetime images, respectively. Denoised lifetime images exhibit notably fewer red pixels, indicating reduced noise and accurate lifetime values compared to noisy lifetime images. The phasors of the selected ROI in the noisy, denoised, and averaged FLIM measurements are presented in Figures 6I–K, respectively Figure 6. Clear identification of the nucleus and mitochondria is evident in the denoised lifetime and phasor images in comparison with the noisy lifetime. The denoised images closely align with the averaged image and exhibit improved SNR values. In Figures 6A–D, the upper series signifies complete FOV of size 512 × 512, while the lower series in Figures 6E–H represents ROI marked by the yellow square of size 125 × 125 of the corresponding upper series images. The results of K-means clustering on the phasors of the noisy, denoised, and averaged FLIM measurements are presented in Figures 6L–N, respectively. The PSNR values of the BPAE samples lifetime images noisy and denoised using DnCNN pre-trained CNNs methods are 13.69 dB and 18.07 dB, respectively. Similarly, the SSIM values of the lifetime images noisy and denoised using DnCNN methods are 0.076 and 0.173, respectively. From the PSNR values, there is an improvement in image quality of 4.38 dB due to DnCNN pre-trained CNNs denoising method. In a previous study, we demonstrated the superior performance of our pre-trained CNNs compared to established image denoising methods like BM3D (Mannam et al., 2022). To further corroborate this finding, we have included qualitative and quantitative comparisons for the same BPAE sample cell in our GitHub repository.

We also conducted imaging on another fixed mouse kidney sample [prepared slide *#*3 (F-24630) by FluoCells], where mouse kidney samples were stained with DAPI (nuclei using blue-fluorescent DNA stain), Alexa Fluor 488 wheat germ agglutinin (highlighting elements of the glomeruli and convoluted tubule using green-fluorescent lectin), and Alexa Fluor 568 phalloidin (abundant in glomeruli and the brush border using red-fluorescent filamentous actin) utilizing our Instant FLIM system (Zhang et al., 2021). The excitation wavelength of the pulse laser was set to 800 nm, and at the sample, the excitation power measured was 3.3 mW. Emission spectra for the above-mentioned three fluorophores in fixed mouse kidney cells can be found at^3^.

**FIGURE 7:**
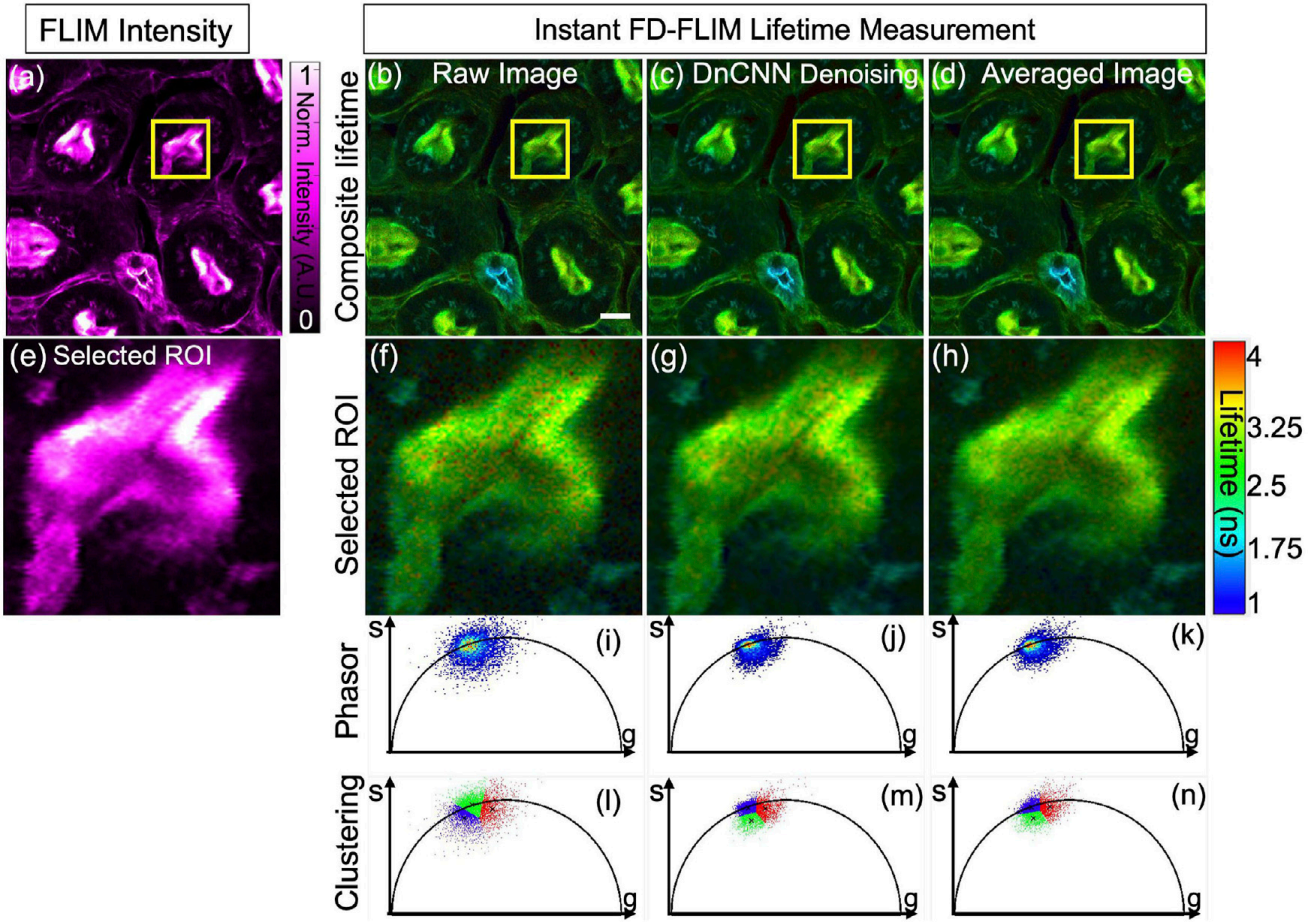
Phasor representation of a mouse kidney fixed sample was analyzed before and after application of pre-trained ML models and the FLIM images were acquired using our instant FD-FLIM system (Zhang et al., 2021). Four images are presented: **(A)** two-photon intensity images, **(B)** noisy images, **(C)** denoised images with our proposed method, and **(D)** mean fluorescence composite lifetime images. Composite lifetime is presented as a Hue Saturation Value (HSV) representation of intensity and lifetime imagery where the brightness and hue of each pixel correspond to the mapped fluorescence intensity and lifetime, respectively. The image size is 512 × 512 pixels with a selective ROI measuring 100 × 100 pixels (marked as yellow box). In **(E–H)**, the obtained intensity and composite lifetime for the noisy, denoised, and averaged images are displayed. The phasors for the noisy, denoised, and averaged lifetime images are displayed in panels **(I–K)**, correspondingly. Red pixels indicate elevated lifetime values, indicating noise in composite lifetime images. Panels **(L–N)** show K-means clustering (K = 3) for the noisy, denoised, and averaged lifetime images, respectively. The excitation wavelength used was 800 nm with sample power set at 3.3 mW and pixel dwell-time of 12 *μ*s. FLIM Intensity image is normalized between 0 to 1 as shown in **(A)**. Scale bar equals 20 μm.

Figure 7A presents the noisy image of the fixed mouse kidney sample, captured through our specialized two-photon FLIM system, displayed as a single-channel image with magenta hot false color. Concurrently, Figures 7B–D demonstrate the composite lifetime of noisy FLIM data, the denoising effect of the pre-trained ML model on the noisy FLIM mouse kidney fixed sample data, and averaged lifetime measurements (averaged over 5 samples within the same FOV), providing high-SNR images for comparison, respectively. Furthermore, Figures 7E–H present the ROIs for the intensity, composite lifetime images of noisy, DnCNN-denoised, and averaged lifetime images, respectively. Notably, denoised lifetime images contain fewer red pixels, indicating diminished noise and precise lifetime values compared to noisy counterparts. Phasors for the noisy, denoised, and averaged FLIM measurements are displayed in Figures 7I–K, respectively. The results of K-means clustering on the phasors of the noisy, denoised, and averaged FLIM measurements are depicted in Figures 7L–N, respectively, illuminating the effectiveness of the denoising process. The PSNR values of the fixed mouse kidney noisy and denoised using DnCNN methods lifetime images are 17.63 dB and 22.10 dB, respectively. Similarly, the SSIM values of the noisy and denoised using DnCNN methods lifetime images are 0.190 and 0.355, respectively. From the PSNR values, there is an improvement in image quality of 4.47 dB due to DnCNN pre-trained CNNs denoising method. Figure 7.

### 3.3 Out-of-distribution samples: high-scattered plant tissues

**FIGURE 8:**
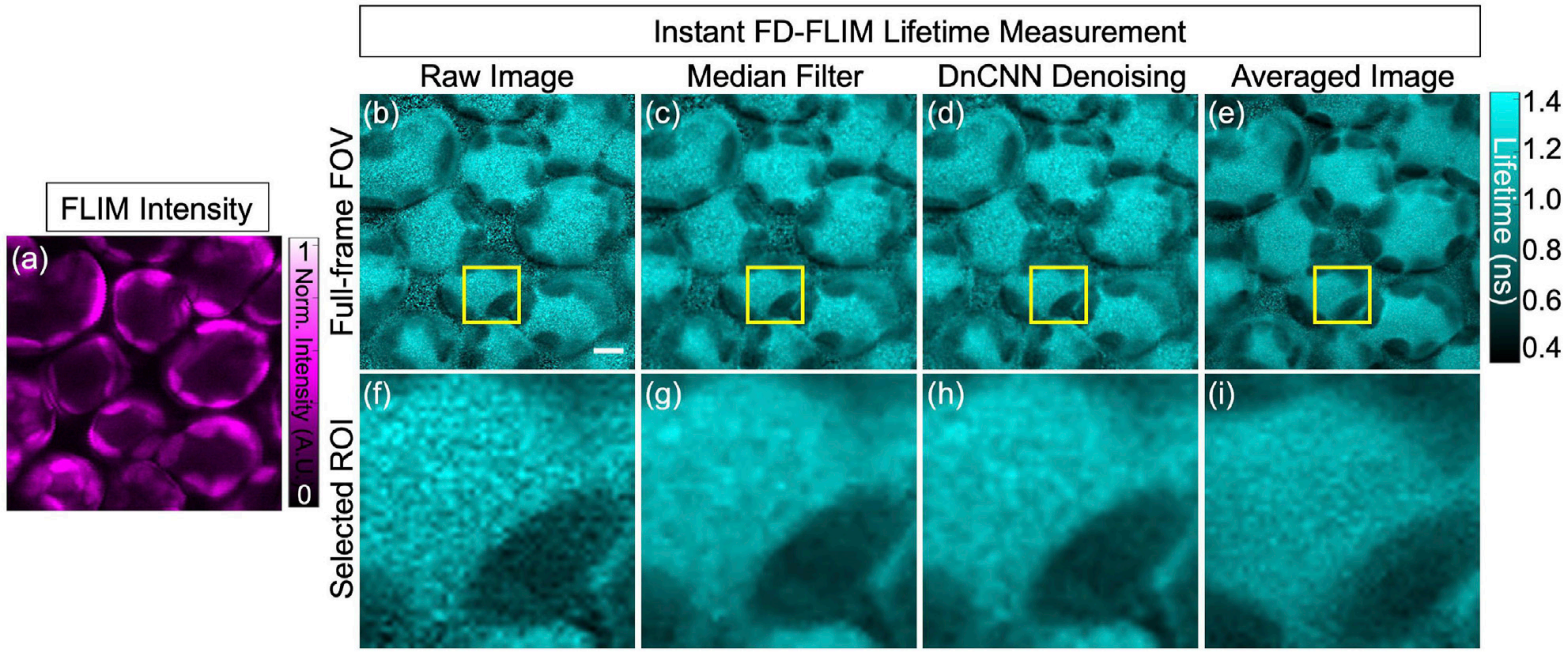
FLIM images of the *in vivo* bean plant spongy mesophyll layer: intensity image **(A)**, lifetime images where raw lifetime **(B)**, median filter denoised lifetime **(C)**, pre-trained DnCNN ML model denoised lifetime **(D)**, and ground-truth (averaged) lifetime image **(E)**, respectively. Likewise, the designated ROI, indicated within the yellow box, is depicted in panels **(F–I)** to display the corresponding selected field-of-view. FLIM intensity and lifetime images are presented in false colors, where each image is a gray-scale image. Excitation wavelength: 800 nm; sample power: 3 mW; pixel dwell-time: 12 *μ*s. FLIM Intensity image is normalized between 0 to 1 as shown in **(A)**. Scale bar, 5 *μ*m.

In our research, deep learning models proficient in training datasets acquired from FLIM systems depict information as 2D sections per channel in the 3D volume across specific time frames. Addressing the challenge of model performance on data that differs from the training set is crucial, especially for complex structures like highly scattering plant tissues. The intricate cellulose cell wall structures in plants cause high optical scattering coefficients, making depth-resolved imaging challenging for conventional microscopy systems. Confocal laser scanning microscopy (CLSM) provides limited depth resolution due to scattering, whereas multi-photon microscopy (MPM) offers deeper penetration. Given this, our approach’s validation on plant samples that differ from the training set is crucial, demonstrating its ability to adapt to new data and generalize the pre-trained models. To assess our pre-trained ML models for lifetime denoising, we used our instant FLIM system (Zhang et al., 2021) for plant tissue imaging. Both phasor axes of FLIM measurements showed an MPG noise distribution, validating the application of our pre-trained denoising method to each *G* and *S* image before lifetime extraction. Notably, the pre-trained DnCNN model denoises images based on single 2D planes, incorporating information from both sides of the plant structure. To obtain ground truth, each illumination plane was imaged a minimum of five times, and the resultant images were averaged to obtain accurate comparisons. Figure 8.

**FIGURE 9:**
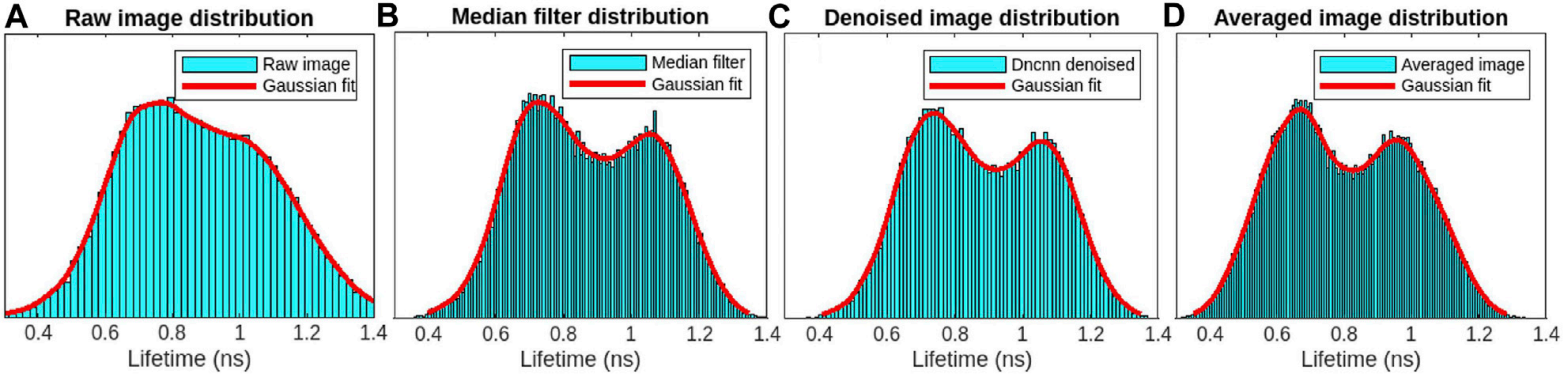
FLIM images of the *in vivo* bean plant spongy mesophyll layer: lifetime image distribution with Gaussian fit for the raw image **(A)**, median filter denoised image **(B)**, DnCNN denoised lifetime using our ImageJ plugin **(C)**, and averaged lifetime image **(D)**, respectively. From the denoised image and averaged images, two-lifetime distributions can be identified, which are missing in the case of the raw lifetime image distribution.

**FIGURE 10:**
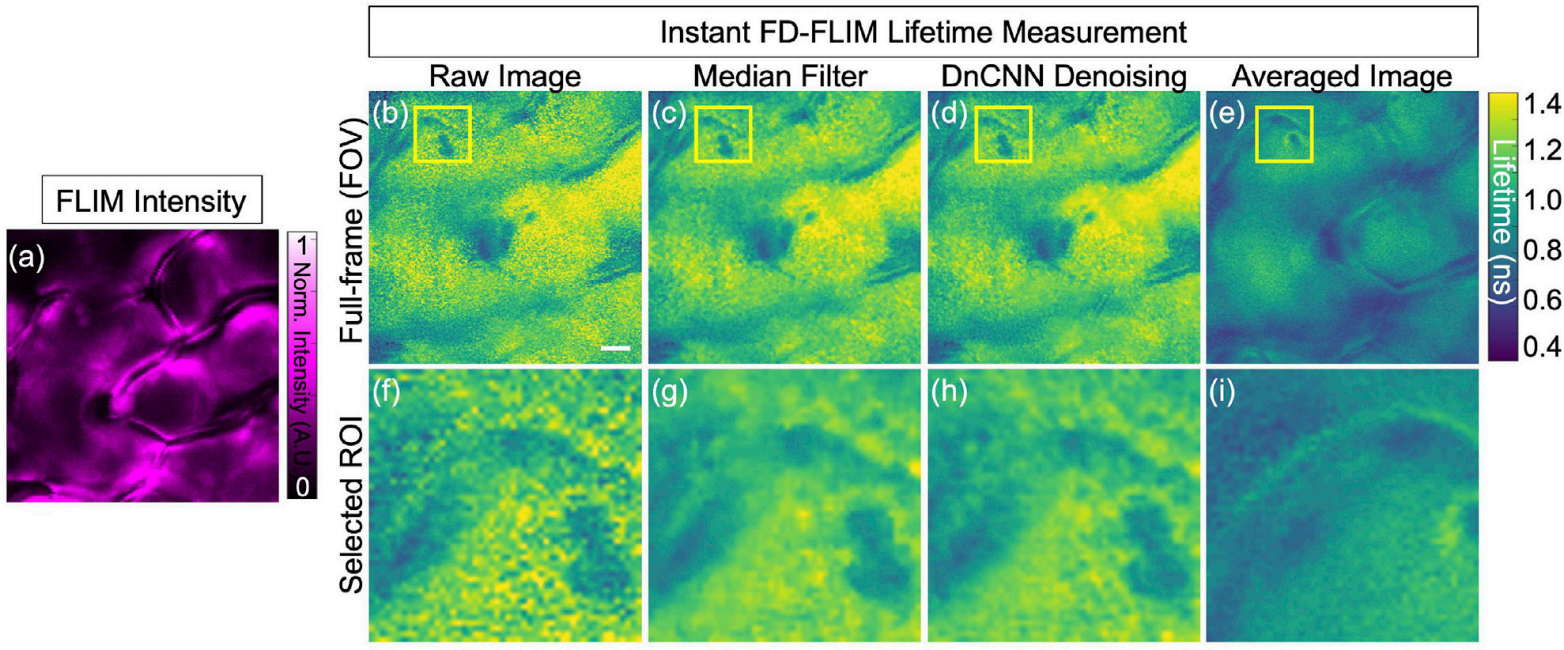
FLIM images of the *in vivo* bean plant Upper epidermis layer: intensity image **(A)**, lifetime images where raw lifetime **(B)**, median filter denoised lifetime **(C)**, DnCNN denoised lifetime using our ImageJ plugin **(D)**, and averaged lifetime image **(E)**, respectively. Likewise, the designated ROI, indicated within the yellow box, is depicted in panels **(F–I)** to display the corresponding selected field-of-view. FLIM intensity and lifetime images are presented in false colors, where each image is a gray-scale image. Excitation wavelength: 800 nm; power: 5 mW; pixel dwell-time: 12 *μ*s. FLIM Intensity image is normalized between 0 to 1 as shown in **(C)**. Scale bar, 5 *μ*m.

Bean plant leaves (grown in our laboratory at room temperature from Ferry-Morse bean seeds) are imaged using our custom-built MPM-FLIM [InstantFLIM (Zhang et al., 2021)] and presented in Figures 8, 10, which represent the top and bottom sides of the active *in vivo* leaf samples of age less than 10 days. Because plants contain autofluorescent molecules, such as chlorophyll and flavonoids, external fluorescent markers or dyes are not needed to image them. Figure 8A shows the fluorescence intensity image (single channel in false color of magenta) of the plant leaf at a depth of 30 *μ*m below the upper epidermis layer. Figure 8B shows the fluorescence lifetime image (single-channel false color in cyan) showing the spongy mesophyll layer. Figure 8C shows the application of a median filter to both components (X-axes and Y-axes) of complex FLIM measurements and the extracted fluorescence lifetime image of a plant leaf. Figure 8D shows the pre-trained DnCNN ML model applied to both orthogonal axes (x- and y-axes) of complex FLIM measurement to produce a denoised fluorescence lifetime image. Figure 8E shows the ground truth lifetime image obtained by averaging in the FLIM complex plane of complex FLIM measurement in the same FOV. Similarly, Figures 8F–I show the smaller field-of-view images of the noisy, median denoised, DnCNN denoised, and averaged lifetime images, respectively. To show the fluorophores in the spongy mesophyll layer, the lifetime histogram of the plant leaf is shown in Figure 9. Figure 9A shows the noisy lifetime image distribution range of 0.3 ns–1.4 ns and cannot clearly show two-lifetime distributions. In addition, each histogram image also contains a Gaussian fit curve of the lifetime image to multiple fluorophore distributions. Figures 9B–D show fluorescence lifetime distributions of applied median filtered image, pre-trained DnCNN ML model denoised fluorescence lifetime image, and averaged fluorescence lifetime images, respectively. The intrinsic autofluorescence of cytosolic structures ranges from 0.75 ns to 1 ns, and chlorophyll ranges from 0.45 ns to 0.75 ns. Our pre-trained DnCNN model clearly shows the lifetime distribution of two fluorescence lifetime decays: chlorophyll and cytosolic structures. The PSNR values of the plant lifetime images denoised using the median filtering and DnCNN methods were 19.05 dB and 19.20 dB, respectively, compared to 17.20 dB for the noisy image. Similarly, the SSIM values of the lifetime images denoised using the median filtering and DnCNN methods were 0.265 dB and 0.274 dB, respectively, compared to 0.162 dB for the noisy image. These results demonstrate the effectiveness of DnCNN pre-trained CNNs denoising method in improving the image quality. Figure 9.

Similarly, Figure 10 shows the upper epidermal layer of the bean plant is imaged using our InstantFLIM system (Zhang et al., 2021). Figure 10A shows the fluorescence intensity image (single channel in the false magenta color) of the upper epidermis layer of the bean plant. Figure 10B shows the fluorescence lifetime image (single-channel false color image in cyan) of the upper epidermis layer. Figure 10C shows the application of a median filter to both phasor axes of the complex FLIM measurements to extract the lifetime image of a plant leaf. Figure 10D shows the pre-trained DnCNN ML model applied to the FLIM phasor space (*G* and *S* images) of the FLIM measurement, producing the denoised lifetime image. Figure 10E shows the ground truth lifetime image obtained by averaging in the FLIM complex plane of the FLIM measurement in the same FOV. The smaller-field-of-view images of the noisy, median-denoised, DnCNN-denoised, and averaged lifetime images are shown in Figure 10F–I, respectively. The intrinsic autofluorescence of cell walls ranges from 0 ns to 0.4 ns, and chlorophyll ranges from 0.75 ns to 1.2 ns. Clearly, our pre-trained DnCNN model shows the lifetime distribution of two fluorescence lifetime decays of cell wall structures and chlorophyll. The PSNR values of the plant upper epidermal-layer lifetime images denoised using the median filtering and DnCNN methods were 11.55 dB and 11.72 dB, respectively, compared to 11.27 dB for the noisy image. Similarly, the SSIM values of the lifetime images denoised using the median filtering and DnCNN methods were 0.285 and 0.345, respectively, compared to 0.265 for the noisy image. These results demonstrate the effectiveness of DnCNN pre-trained CNNs denoising method in improving the image quality. Figure 10.

**FIGURE 11:**
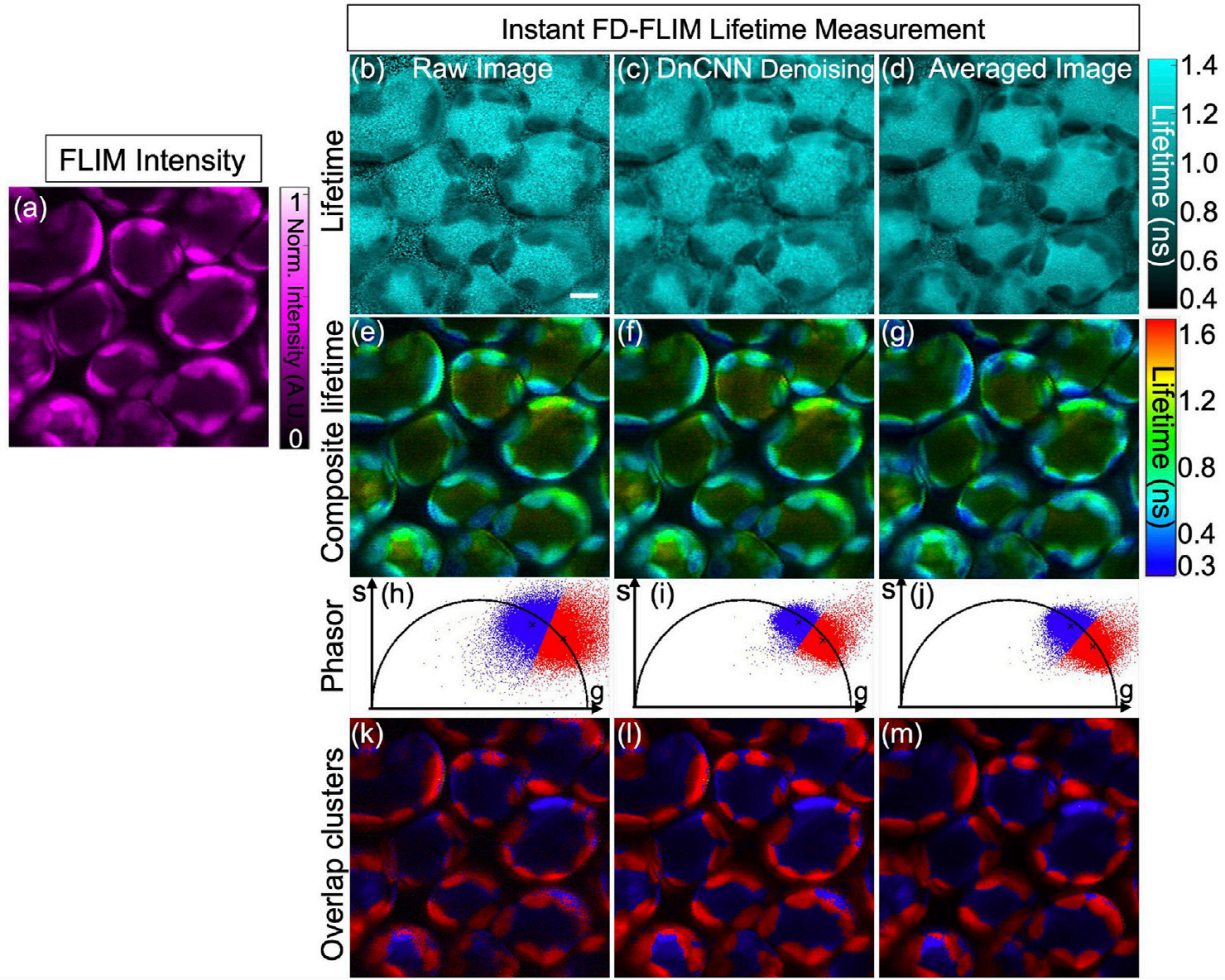
FLIM images of the *in vivo* bean plant spongy mesophyll layer: intensity image **(A)**, lifetime images where raw lifetime **(B)**, DnCNN denoised lifetime using our ImageJ plugin **(C)**, and averaged lifetime image **(D)**, respectively. FLIM intensity and lifetime images are presented in false colors, where each image is a gray-scale image. Composite lifetime images are shown in **(E–G)**, respectively, in the HSV format where value (pixel brightness) is mapped as fluorescence intensity and hue (color) is mapped as fluorescence lifetime. Phasor diagrams with K-means clustering with 2 clusters (k = 2) are shown in **(H–J)**, respectively. Overlap clusters of the lifetime images are shown in **(K–M)**, respectively. FLIM Intensity image is normalized between 0 to 1 as shown in **(A)**. Excitation wavelength: 800 nm; power: 3 mW; pixel dwell-time: 12 *μ*s. Scale bar, 5 *μ*m.

Once the phasor axes (*G* and *S* images) of complex FLIM measurement data are denoised using the pre-trained DnCNN model, the denoised phasor is extracted, as shown in Figure 11. In addition, the phasor segments of the phasor are extracted using the K-means clustering method. Figure 11A shows the single-channel gray-scale image in false magenta color intensity of the *in vivo* bean plant leaf spongy mesophyll layer. Figure 11B–D show the single channel gray-scale image in false cyan color raw, DnCNN denoised, and averaged lifetime images, respectively. Figure 11E–G show the false-color composite HSV lifetime image, where value (pixel brightness) represents fluorescence intensity and hue (color) represents fluorescence lifetime of the raw, DnCNN-denoised, and averaged lifetime images, respectively. Figure 11H–J show the phasors of the raw, DnCNN denoised, and averaged phasors, respectively. Figure 11H shows the phasor with a larger noise distribution, while the denoised phasor in Figure 11I shows reduced noise with improved SNR. In addition, the phasors are divided into two groups to represent the chlorophyll and cytosolic structures, as shown in red and blue colors, respectively. Figure 11K–M show clusters of the two fluorophores in the raw, DnCNN-denoised, and averaged lifetime clusters, where the blue color pixels represent the chlorophyll and red color pixels represent the cytosolic structures. Figure 11.

Finally, FLIM lifetime denoising results using the Noise2Noise pre-trained CNN model are provided in GitHub^4^. The outcomes disclosed in this study are available publicly and can be accessed through the same GitHub repository.

### 3.4 Limitation of pre-trained CNNs on lifetime image denoising

Our pre-trained ML models consistently perform well on unseen fluorescence intensity image structures and samples, demonstrating their ability to generalize and avoid over-fitting. This means they can effectively denoise fluorescence lifetime images with various structures and samples (such as fluorescence nanobeads, etc.,) that differ from those during training. However, when it comes to noise distribution, denoising lifetime images using pre-trained CNNs trained on fluorescence intensity images can be challenging due to the complex noise distribution of fluorescence lifetime images. Our method addresses this challenge by employing the complex phasor representation of FLIM images, where each component of complex FLIM measurement provides a noise distribution of the Skellam distribution that can be approximated to Gaussian noise and is compatible with pre-trained CNNs noise distribution of MPG noise.

Recent FLIM image acquisition methods often produce multidimensional data (spatial (*XYZ*), temporal (*T*), and multiple channels at different emission filters (*λ*_1_, *λ*_2_), which are typically presented in 2D sectional images of complex FLIM measurement. The noise in each 2D sectional image follows the Skellam distribution. Further performance improvements could be achieved by training models directly on these 2D images. This would require a large training dataset, which can be difficult and expensive to acquire, especially for *in vivo* imaging. However, this approach has the potential to significantly improve fluorescence lifetime phasor denoising. Currently, our approach is limited to processing 3D sections of FLIM data, meaning that multidimensional FLIM data requires pre-processing into 3D sections of *XYZ* (entire volume at each discrete time interval) or *XYT* (3D stack of 2D planes at different time intervals) in each acquisition channel before applying our method. Following the application of our image denoising method, the denoised results must be combined as part of a post-processing step. Finally, it is always recommended to check if the generated denoised lifetime images have any artifacts using the existing quantitative metrics such as the phasor position of the fluorophores to indicate accurate lifetime values.

## 4 Conclusion

To overcome the challenges of slow data capture (longer time for imaging), low SNR, and costly setups in fluorescence lifetime imaging microscopy (FLIM), we introduce Instant FLIM, a unique, rapid processing FLIM instrument using real-time signal processing and commercial analog processing devices to provide fluorescence intensity, lifetime, and phasors for multi-dimensional FLIM data, including 2-D (*XY* plane), 3-D (*XYZ* with *Z*: depth), or 4-D (*XYZT* with *Z*: depth, *T*: time) *in vivo* imaging. This paper demonstrated the utilization of pre-trained deep learning-based image denoising models to remove noise from complex FLIM measurements before extracting the sample fluorescence lifetime and sample phasor plot. The denoised phasor shows a qualitative improvement in SNR in FLIM images. Subsequent application of denoised phasor data to K-means clustering segmentation reveals distinct segments corresponding to different fluorophores. Our demonstrated method has been rigorously tested on diverse samples, including *in vivo* mouse kidney tissue, fixed BPAE samples, fixed mouse kidney samples, and highly scattering plant samples, demonstrating its efficacy in enhancing the detection of key biological structures in FLIM applications. Overall, the combination of Instant FLIM and pre-trained ML models for denoising offers a fast and accurate solution for fluorescence image denoising, yielding high SNR and accurate segmentation.

## Data Availability

The original contributions presented in the study are included in the article are available in the GITHub location: https://github.com/ND-HowardGroup/FLIM_Denoising_using_Pretrained_CNNs, further inquiries can be directed to the corresponding author.

## Footnotes

1
https://github.com/ND-HowardGroup/Instant-Image-Denoising/tree/master/Plugins/Image_Denoising_Plugins_Journal/Plugin_Targets

2
https://www.thermofisher.com/order/catalog/product/F36924

3
https://www.thermofisher.com/order/catalog/product/F24630

4
https://github.com/ND-HowardGroup/FLIM_Denoising_using_Pretrained_CNNs.git

## References

[B1] Chang C.-W. Sud D. Mycek M.-A. (2007). Fluorescence lifetime imaging microscopy. Methods Cell Biol. 81, 495–524. 10.1016/s0091-679x(06)81024-1 17519182

[B2] contributors W. (2004). Skellam distribution. Available at: https://en.wikipedia.org/wiki/Skellam_distribution .

[B3] Dabov K. Foi A. Katkovnik V. Egiazarian K. (2007). Image denoising by sparse 3-D transform-domain collaborative filtering. IEEE Trans. Image Process. 16, 2080–2095. 10.1109/tip.2007.901238 17688213

[B4] Datta R. Heaster T. M. Sharick J. T. Gillette A. A. Skala M. C. (2020). Fluorescence lifetime imaging microscopy: fundamentals and advances in instrumentation, analysis, and applications. J. Biomed. Opt. 25, 071203. 10.1117/1.jbo.25.7.071203 32406215 PMC7219965

[B5] Digman M. A. Gratton E. Marcu L. French P. Elson D. (2014). The phasor approach to fluorescence lifetime imaging: exploiting phasor linear properties. Fluoresc. Lifetime Spectrosc. Imaging, 235–248. 10.1201/b17018-14

[B6] Gabriel P. R. (2008). SSIM: a java plugin in ImageJ. Available at: https://imagej.nih.gov/ij/plugins/ssim-index.html .

[B7] Goodfellow I. Bengio Y. Courville A. (2016). Deep learning. MIT press.

[B8] Griffin T. F. (1992). Distribution of the ratio of two Poisson random variables. Available at: https://ttu-ir.tdl.org/bitstream/handle/2346/59954/31295007034522.pdf .

[B9] Hore A. Ziou D. (2010). “Image quality metrics: PSNR vs. SSIM,” in 2010 20th international conference on pattern recognition (IEEE), 2366–2369.

[B10] Jain A. K. (2010). Data clustering: 50 years beyond K-means. Pattern Recognit. Lett. 31, 651–666. 10.1016/j.patrec.2009.09.011

[B11] Lehtinen J. Munkberg J. Hasselgren J. Laine S. Karras T. Aittala M. (2018). Noise2Noise: learning image restoration without clean data. *Proc. Mach. Learn. Res.* (PMLR) 80, 2965–2974.

[B12] MacQueen J. (1967). “Some methods for classification and analysis of multivariate observations,” in Proceedings of the fifth Berkeley symposium on mathematical statistics and probability, Oakland, CA, USA 1 (14), 281–297.

[B13] Mannam V. (2022). Overcoming fundamental limits of three-dimensional *in vivo* fluorescence imaging using machine learning. 10.7274/5x21td99n58

[B14] Mannam V. Howard S. (2023). Small training dataset convolutional neural networks for application-specific super-resolution microscopy. J. Biomed. Opt. 28, 036501. 10.1117/1.jbo.28.3.036501 36925620 PMC10013193

[B15] Mannam V. Kazemi A. (2020). Performance analysis of semi-supervised learning in the small-data regime using VAEs. *arXiv preprint arXiv:2002.12164* .

[B16] Mannam V. Zhang Y. Yuan X. Hato T. Dagher P. C. Nichols E. L. (2021). Convolutional neural network denoising in fluorescence lifetime imaging microscopy (FLIM). In Multiphoton Microscopy in the Biomedical Sciences XXI (International Society for Optics and Photonics (SPIE Photonics West)), vol. 11648, 116481C

[B17] Mannam V. Zhang Y. Yuan X. Ravasio C. Howard S. S. (2020a). Machine learning for faster and smarter fluorescence lifetime imaging microscopy. J. Phys. Photonics 2, 042005. 10.1088/2515-7647/abac1a

[B18] Mannam V. Zhang Y. Zhu Y. Howard S. (2020b). Instant image denoising plugin for ImageJ using convolutional neural networks *Microscopy Histopathology and Analytics* (Optical Society of America). MW2A–3.

[B19] Mannam V. Zhang Y. Zhu Y. Nichols E. Wang Q. Sundaresan V. (2022). Real-time image denoising of mixed Poisson–Gaussian noise in fluorescence microscopy images using ImageJ. Optica 9, 335–345. 10.1364/optica.448287

[B20] MATLAB (2019). 9.7.0.1190202 (R2019b) (natick, Massachusetts: the MathWorks inc.).

[B21] Nehme E. Weiss L. E. Michaeli T. Shechtman Y. (2018). Deep-STORM: super-resolution single-molecule microscopy by deep learning. Optica 5, 458–464. 10.1364/optica.5.000458

[B22] Rueden C. Schmidt D. Wilhelm B. (2017). ImageJ tensorflow library. Available at: https://github.com/imagej/imagej-tensorflow .

[B23] Sage D. (2017). ImageJ’s plugin to assess the quality of images. Available at: http://bigwww.epfl.ch/sage/soft/snr/ .

[B24] von Chamier L. Laine R. F. Jukkala J. Spahn C. Krentzel D. Nehme E. (2021). Democratising deep learning for microscopy with ZeroCostDL4Mic. Nat. Commun. 12, 2276–2293. 10.1038/s41467-021-22518-0 33859193 PMC8050272

[B25] Wang P. Hecht F. Ossato G. Tille S. Fraser S. Junge J. (2021). Complex wavelet filter improves FLIM phasors for photon starved imaging experiments. Biomed. Opt. Express 12, 3463–3473. 10.1364/boe.420953 34221672 PMC8221945

[B26] Weigert M. Schmidt U. Boothe T. Müller A. Dibrov A. Jain A. (2018). Content-Aware Image Restoration: pushing the limits of fluorescence microscopy. Nat. Methods 15, 1090–1097. 10.1038/s41592-018-0216-7 30478326

[B27] Zhang K. Zuo W. Chen Y. Meng D. Zhang L. (2017). Beyond a Gaussian denoiser: residual learning of deep CNN for image denoising. IEEE Trans. Image Process. 26, 3142–3155. 10.1109/tip.2017.2662206 28166495

[B28] Zhang Y. Guldner I. H. Nichols E. L. Benirschke D. Smith C. J. Zhang S. (2021). Instant FLIM enables 4D *in vivo* lifetime imaging of intact and injured zebrafish and mouse brains. Optica 8, 885–897. 10.1364/optica.426870 PMC11759494 39867356

[B29] Zhang Y. Hato T. Dagher P. C. Nichols E. L. Smith C. J. Dunn K. W. (2019). Automatic segmentation of intravital fluorescence microscopy images by K-means clustering of FLIM phasors. Opt. Lett. 44, 3928–3931. 10.1364/ol.44.003928 31415514

